# Molecular Characterization of cagA and vacA Virulence Genes in Helicobacter pylori Isolates From Patients With Gastroduodenal Diseases: A Cross-Sectional Study

**DOI:** 10.7759/cureus.100778

**Published:** 2026-01-04

**Authors:** Sushrita Mohanty, Nirmala Poddar, Ipsa Mohapatra, Rajesh K Dash, Sushant K Sethi, Jagadananda Jena, Dipti Pattnaik

**Affiliations:** 1 Microbiology, Hi-Tech Medical College, Bhubaneswar, IND; 2 Microbiology, Kalinga Institute of Medical Sciences, Bhubaneswar, IND; 3 Community Medicine, Kalinga Institute of Medical Sciences, Bhubaneswar, IND; 4 Institute of Gastroenterology, Apollo Hospitals, Bhubaneswar, IND

**Keywords:** cytotoxin-associated gene a, gastrointestinal, helicobacter pylori, upper gastrointestinal disorders, vaca

## Abstract

Background

*Helicobacter pylori* is an important gastric pathogen linked to several upper gastrointestinal (UGI) conditions, including gastritis, peptic ulcer disease, and gastric cancers. Its ability to cause disease is largely driven by major virulence factors, such as the cytotoxin-associated gene A (cagA) and the vacuolating cytotoxin gene (vacA). This study aimed to determine the prevalence of *H. pylori* among patients presenting with UGI symptoms using both phenotypic and molecular methods, and to identify the presence of cagA and vacA genes in the isolates.

Materials and methods

Patients with symptomatic GI complaints who underwent UGI endoscopy were enrolled. Four gastric biopsy specimens were collected from each patient in brain-heart infusion medium and sent from the medical gastroenterology division for microbiological and molecular testing. Identification of *H. pylori* was performed using standard culture techniques with Skirrow Campylobacter medium and growth supplements. Molecular detection was carried out using polymerase chain reaction (PCR). DNA was extracted using Amp Ready reagent and stored at -20°C until analysis. Data were collected using a structured proforma and analyzed with Epi Info version 7.3.2 (Centers for Disease Control and Prevention (CDC), Atlanta, GA, USA). Categorical variables were summarized as percentages with 95% confidence intervals (CI). Associations were assessed using the chi-square test or Fisher’s exact test, with p ≤ 0.05 considered statistically significant.

Results

Among the 250 gastric biopsy samples processed, *H. pylori* grew on culture in 38 samples (15.2%). PCR targeting the *ureB* gene detected *H. pylori* DNA in 36 samples (14.4%). Of these PCR-positive isolates, 30 (83.3%) carried both the cagA and vacA virulence genes. Genotyping showed that the vacA s1 and m1 alleles frequently coexisted, with the s1m1 genotype being the most common and associated with higher virulence.

Conclusion

PCR-based testing proved more sensitive and efficient for detecting *H. pylori* and its key virulence markers compared to conventional culture methods. Incorporating molecular techniques into routine diagnostic workflows may facilitate earlier and more accurate identification of high-risk strains.

## Introduction

*Helicobacter pylori* is a spiral-shaped, gram-negative bacterium that grows under microaerophilic conditions and is known to colonize the gastric mucosa of nearly half of the world’s population. It is associated with several gastrointestinal (GI) disorders, including chronic gastritis, acid peptic disease, and gastric ulcers, and is also recognized as a trigger for various types of gastric lymphomas [1]. Due to its strong link with peptic ulcer disease and gastric cancer, the International Agency for Research on Cancer (IARC), a branch of the World Health Organization (WHO), has designated *H. pylori* as a Group I carcinogen [2]. Differences in clinical outcomes among infected individuals are largely driven by bacterial virulence factors, particularly the vacuolating cytotoxin gene (vacA), the cytotoxin-associated gene (cagA), and the urease (ure) gene [3]. The urease enzyme helps protect *H. pylori* from the acidic gastric environment by converting urea into ammonia. The VacA protein, a 95-kDa highly immunogenic toxin encoded by the vacA gene, plays a major role in gastroduodenal disease severity. Found in most *H. pylori* strains, this pore-forming toxin damages gastric epithelial cells by inducing vacuole formation, creating membrane channels, disrupting cytoskeleton-dependent functions, and promoting apoptosis. It also influences host immune responses [3,4]. The vacA gene includes two variable regions: the signal (s) region, with subtypes s1a, s1b, and s2, and the middle (m) region, with alleles m1 and m2. Strains carrying s1/m1 and s1/m2 combinations tend to produce moderate to high cytotoxic activity, whereas s2/m2 strains produce little to none [3,4].

The cagA gene, located within the cag pathogenicity island (cagPAI), encodes the CagA oncoprotein. After being delivered into gastric epithelial cells through the type IV secretion system, CagA interacts with host signaling proteins, most notably the tyrosine phosphatase SHP-2. This interaction drives cytoskeletal changes and morphological abnormalities that contribute to gastric carcinogenesis [5].

The aim of this study was to determine the prevalence of *H. pylori* in patients presenting with dyspepsia and to assess the pathogenic potential of the isolates by detecting key virulence genes, cagA and vacA.

## Materials and methods

This study was conducted in the Department of Microbiology, Kalinga Institute of Medical Sciences (KIMS), Bhubaneswar, in collaboration with the National Institute of Cholera and Enteric Diseases (NICED), Kolkata. The research was carried out over a period of nearly two years (November 2013-August 2015). Ethical approval was obtained from the Institutional Ethics Committee (IEC approval number: KIMS/ETHICS/513/2013). Written informed consent was collected from all participants in accordance with the established ethical guidelines.

Study participants

Inclusion Criteria

Adults aged 18-75 years, of either sex, patients presenting with dyspeptic symptoms, and individuals attending the gastroenterology outpatient department (OPD) at KIMS during the study period were included.

Exclusion Criteria

Patients without dyspeptic symptoms, individuals aged <18 years or >75 years, a history of proton pump inhibitor (PPI) or antibiotic use within the previous two weeks, and patients with a known diagnosis of carcinoma were excluded from the study.

Sample collection

During endoscopy, four gastric biopsy samples were collected from the antral and body regions of the stomach. Each sample was processed separately for the rapid urease test, bacterial culture, and histopathological examination. For histopathology, biopsy specimens were fixed in 10% buffered formalin, routinely processed, and stained with hematoxylin and eosin (H&E) and Giemsa stains. In cases where *H. pylori* was successfully isolated on culture, the virulence genes ureB, cagA, and vacA were further identified using polymerase chain reaction (PCR).

Transport of specimen

Collected biopsy samples were placed in 0.6 mL of Brucella broth (Difco Laboratories) containing 15% glycerol and transported on ice immediately after collection. Samples were stored at -70°C until further processing for bacterial culture.

Microbiological processing

Each biopsy specimen was tested for urease activity using Christensen’s urea broth and incubated at 37°C for one hour, with appropriate positive and negative controls. For culture, biopsy samples were crushed between two sterile glass slides, and the minced tissue was inoculated onto freshly prepared Skirrow’s Campylobacter selective medium containing brucella agar base (Himedia MO74) and Campylobacter growth supplements (Himedia FD009 and FD008) with defibrinated horse serum (Himedia RM1239), as well as chocolate agar as a non-selective medium [6].

The inoculated plates were incubated at 37°C under microaerophilic conditions and observed for 3-7 days for bacterial growth. Colonies suggestive of *H. pylori* appeared small (<2 mm), circular, translucent, and grayish. Suspected isolates were further confirmed by Gram staining (showing Gram-negative curved bacilli), motility testing, and positive catalase, oxidase, and urease reactions, in correlation with histopathological findings.

Isolation and identification of *H. pylori*

Genomic DNA was extracted from cultured *H. pylori* isolates using the cetyltrimethylammonium bromide (CTAB) method. In this process, the bacterial cells were first lysed, and the DNA was purified with phenol-chloroform extraction, followed by ethanol precipitation. The final DNA pellets were dissolved in nuclease-free water and stored at -20°C until they were used for PCR.

PCR was then carried out using specific primers targeting several key *H. pylori* genes. The ureB gene was amplified to confirm the identity of the isolates. The cagA gene was tested to check for the presence of the cytotoxin-associated gene A, which is linked to higher virulence. The vacA gene was also analyzed to identify its signal (s) and middle (m) region alleles, since different combinations of these alleles can influence toxin activity.

Each PCR reaction had a total volume of 25 µL and included standard PCR buffer (Genei, Bangalore, India), 1 U of Taq DNA polymerase, 1.5 mM MgCl₂, 0.25 mM of each dNTP, 25 pmol of each primer, and about 50 ng of template DNA. Amplification was performed using an Eppendorf Mastercycler with the following conditions: 35 cycles of denaturation at 95°C for 1 minute, annealing at 55°C for 1 minute, and extension at 72°C for 1 minute. A final extension step was carried out at 72°C for 10 minutes.

PCR products were separated on a 1.5% agarose gel stained with ethidium bromide (or another safe nucleic acid dye). After electrophoresis, the gels were examined under UV light, and a 100-bp DNA ladder was used to verify the sizes of the amplified fragments.

DNA extracted from the *H. pylori* reference strain ATCC 26695 was used as a positive control in every PCR run. Reactions set up without template DNA were included as negative controls to ensure that there was no contamination. The primer sequences [7] and the genes they target are listed in Table 1, which summarizes all published primers used in this study.

**Table 1:** Published primers employed in this study

Target gene	Primer	Sequence (5’-3’)	Amplicon size (bp)
ureB	UreBF	CGTCCGGCAATAGCTGCCATAGT	480
	UreBR	GTAGGTCCTGCTACTGAAGCCTTA	
cagA	CagA5cF	GTTGATAACGCTGTCGCTTCA	350
	CagA3cR	GGGTTGTATGATATTTTCCATAA	
vacA (s region)	vacAs1F	ATGGAAATACAACAAACACAC	s1-259/s2-286
	vacAs1R	CTGCTTGAATGCGCCAAAC	
vacA (m region)	vacAmF	CAATCTGTCCAATCAAGCGAG	m1-567/m2-642
	vacAmR	GCGTCAAAATAATTCCAAGG	

Data analysis 

Data collected were coded and entered into a Microsoft Excel spreadsheet (Microsoft Corp., Redmond, WA, USA). Statistical analysis was performed using Epi Info version 7.3.2 (Centers for Disease Control and Prevention (CDC), Atlanta, GA, USA). Categorical variables were summarized as percentages with corresponding 95% confidence intervals (CI) and compared using either the chi-square test or Fisher’s exact test, depending on appropriateness. A p-value ≤ 0.05 was considered statistically significant.

## Results

A total of 250 gastric biopsy samples were collected from patients with dyspepsia and processed in the gastroenterology department. Of these, 38 samples (15.2%) were positive for *H. pylori* by culture, while 36 samples (14.4%) were confirmed positive by PCR targeting the ureB gene. Infection was more common in males (71%) than females (28%), and the highest prevalence was observed in the 41-50 years age group (44.7%) (Table 2).

**Table 2:** Baseline demographic profile of the study population (N = 250)

Demographic variables	Total (N = 250)	*H. pylori-*positive (n = 38)	*H. pylori-*negative (n = 212)	Odds ratio (95% confidence interval)	p-value
Male	154	27	127	1.64 (0.77-3.40)	0.19
Female	96	11	85		
<20 years	9	1	8	0.69 (0.08-5.67)	0.73
21-30 years	30	2	28	0.37 (0.08-1.60)	0.18
31-40 years	52	9	43	3.17 (1.75-5.73)	0.0001
41-50 years	88	17	71	1.61 (0.79-3.24)	0.18
51-60 years	35	5	30	0.91 (0.33-2.54)	0.87
61-70 years	19	3	16	1.05 (0.29-3.79)	0.94
>70 years	17	1	16	0.33 (0.04-2.57)	0.29

Among the 38 *H. pylori* strains isolated, 23 were from patients with duodenal ulcers, 5 from gastric ulcer patients, 8 from patients with gastritis, and 2 from patients with gastric carcinoma (Table 3).

**Table 3:** Endoscopic findings among study participants with Helicobacter pylori positivity (N = 38)

Endoscopic findings	Total (N = 250)	*H. pylori-p*ositive (n = 38)	*H. pylori-*negative (n = 212)	Odds ratio (95% CI)	p-value
Gastric carcinoma	22	2	20	0.53 (0.12-2.38)	0.41
Duodenal ulcer	85	23	62	3.71 (1.82-7.58)	0.0003
Gastric ulcer	43	5	38	0.69 (0.25-1.89)	0.48
Gastritis	61	8	53	0.80 (0.35-1.85)	0.60

Among the *H. pylori*-positive patients, the most common symptoms were dyspepsia (24.1%), followed by acid regurgitation (23.2%), indigestion (21.4%), loss of appetite (14.2%), and epigastric tenderness (10.5%) (Table 4).

**Table 4:** Distribution of common symptoms among the patients (N = 250) *Chi-square test. **Fisher’s exact test.

Symptom	Total cases (n)	*H. pylori-*positive (n = 38)	*H. pylori-*negative (n = 212)	Test statistic	df	p-value
Epigastric pain	38	4 (10.53%)	34 (89.47%)	0.39	1	0.53*
Dyspepsia	29	7 (24.14%)	22 (75.86%)	0.17	1	0.15**
Acid regurgitation	86	20 (23.26%)	66 (76.74%)	5.68	1	0.01*
Loss of appetite	14	2 (14.29%)	12 (85.71%)	1.00	1	0.92**
Indigestion	14	3 (21.43%)	11 (78.57%)	0.45	1	0.50**

The association of alcohol consumption, smoking, and diabetes with *H. pylori* infection was evaluated. Alcohol consumption was significantly associated with infection, conferring 2.87 times higher odds of acquiring *H. pylori* (p = 0.02). Smoking (p = 0.42) and diabetes (p = 0.26) were not significantly associated. Various risk factors are summarized in Table 5.

**Table 5:** Analysis of risk factors associated with Helicobacter pylori infection PPI: proton pump inhibitor, HTN: hypertension.

Risk factors	Total (N = 250)	*H. pylori*-positive (n = 38)	*H. pylori-*negative (n = 212)	Odds ratio (95% confidence interval)	p-value
Alcoholic	10	08	02	28 (5.68-138.14)	0.001
Non-alcoholic	240	30	210		
Smoker	97	17	80	1.33 (0.67-2.68)	0.42
Nonsmoker	153	21	132		
Diabetics	32	07	25	1.68 (0.67-4.2)	0.26
Non-diabetics	218	31	187		
HTN	102	14	88	0.82 (0.40-1.68)	0.59
Non-HTN	148	24	124		

Of the 250 gastric biopsy samples tested, 177 (70.8%) were positive by rapid urease test (RUT), 38 (15.2%) were positive by culture, and 66 (26.4%) were positive on histopathology. Using culture as the gold standard, a positive prevalence of 100% was recorded (Table 6).

**Table 6:** Specificity and sensitivity of various diagnostic tests in detecting Helicobacter pylori PCR: polymerase chain reaction, ureB gene: urease B gene, PPV: positive predictive value, NPV: negative predictive value.

Lab tests	Positive samples, N (%)	Sensitivity	Specificity	PPV	NPV
Rapid urease test	177/250 (70.80)	100.00%	34.43%	21.47%	100.00%
Histology	66/177 (37.30)	100.00%	86.79%	57.58%	100.00%
PCR (ureB gene)	36/38 (94.70)	94.74%	100.00%	94.74%	99.07%

**Figure 1:**
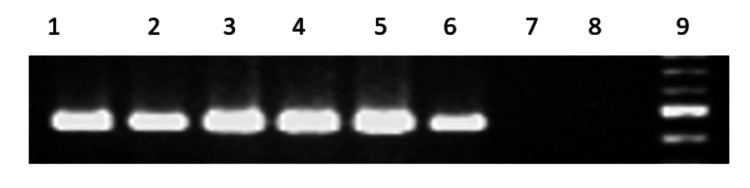
PCR amplification of the ureB gene of Helicobacter pylori Lane 1: positive control. Lanes 2-7: clinical isolates. Lane 8: *Escherichia coli* DNA (negative control). Lane 9: 100-bp DNA ladder (molecular weight marker). The expected amplicon size was 480 bp. Out of the 38 culture-positive *H. pylori* isolates, 36 (94.7%) were ureB-positive, while 2 negative samples were excluded from further analysis.

**Figure 2:**
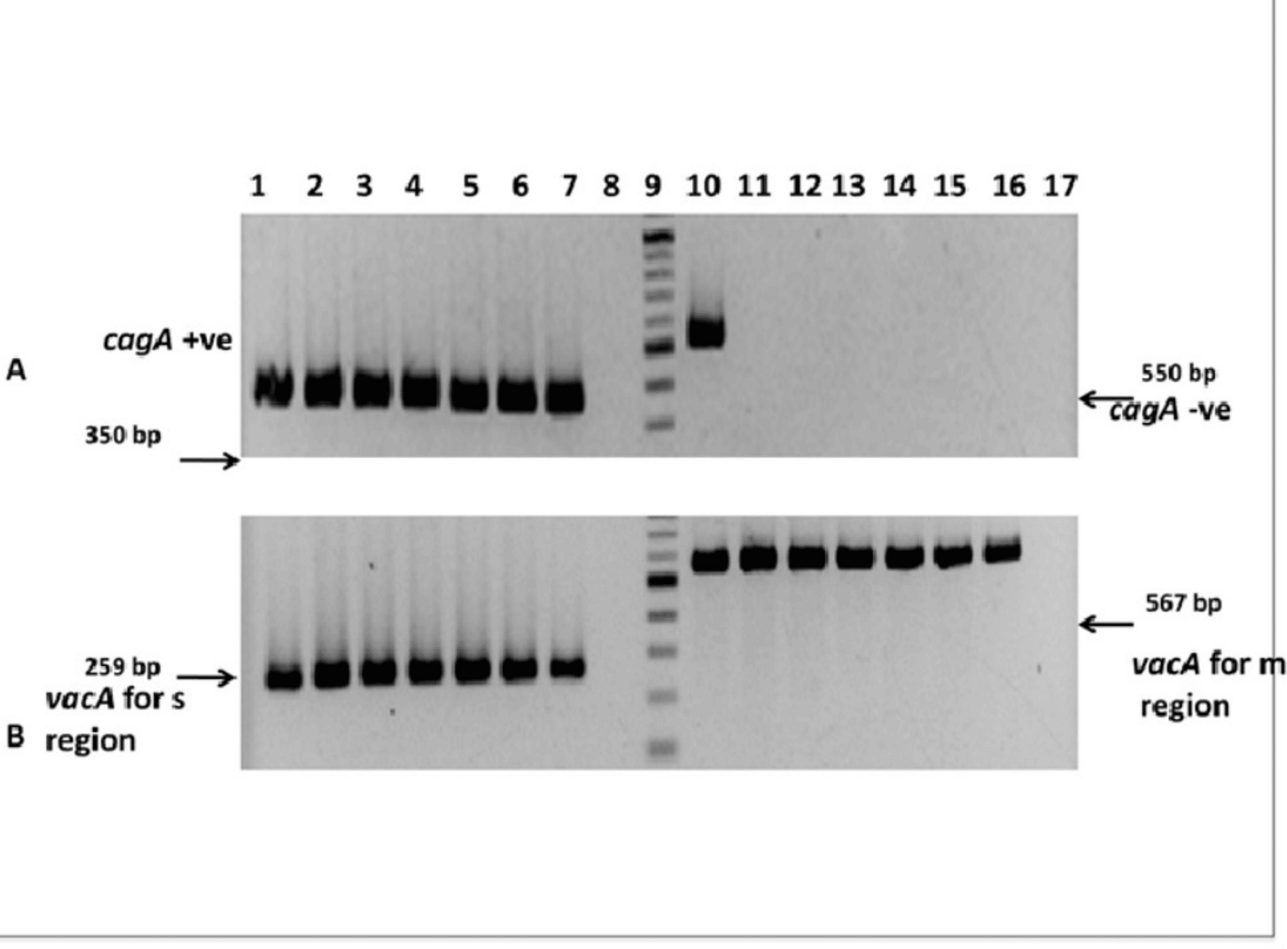
Molecular characterization of virulence genes (cagA and vacA) (A) Amplification of the cagA gene showing a 350 bp product for cagA-positive isolates and a 550 bp product for cagA-negative isolates. (B) Amplification of the vacA gene showing a 259 bp product for the s-region and a 567 bp product for the m-region. Out of the 36 ureB-positive isolates, 30 (83.3%) were positive for both cagA and vacA genes. The toxic allele s1m1 of vacA was present in all vacA-positive strains. cagA: cytotoxin-associated gene A, vacA: vacuolating cytotoxin gene.

Of the 250 samples tested, 36 (14.4%) were positive for *H. pylori* by PCR targeting the ureB gene (Table 6, Figure 1). Among these 36 PCR-positive isolates, 30 (83.3%) were positive for both cagA and vacA genes (Table 7, Figure 2).

**Table 7:** Association between the cytotoxin-associated gene A (cagA) and vacuolating cytotoxin gene (vacA) with endoscopic findings among 36 Helicobacter pylori-positive patients

Endoscopic finding	Total cases (n)	cagA/vacA-positive (n = 30)	cagA/vacA-negative (n = 6)	Test statistic	df	p-value
Gastric carcinoma	2	2 (100.00%)	0 (0.00%)	1.00	1	1.00**
Duodenal ulcer	22	19 (86.36%)	3 (13.64%)	0.02	1	0.89*
Gastric ulcer	5	4 (80.00%)	1 (20.00%)	1.00	1	1.00**
Gastritis	7	2 (28.57%)	5 (71.43%)	0.0003	1	0.07**

The cagA and vacA virulence genes were detected in 100% of gastric carcinoma cases, 86.3% of duodenal ulcer cases, 80% of gastric ulcer cases, and 71.4% of gastritis cases (Table 8).

**Table 8:** Association of cagA and vacA virulence genes with endoscopic findings in Helicobacter pylori strains (n = 36) *Chi-square test. **Fisher’s exact test. cagA: cytotoxin-associated gene A, vacA: vacuolating cytotoxin gene.

Endoscopic finding	Total cases (n)	cagA/vacA-positive (n = 30)	cagA/vacA-negative (n = 6)	Test Statistic	df	p-value
Gastric carcinoma	2	2 (100.00%)	0 (0.00%)	1.00	1	1.00**
Duodenal ulcer	22	19 (86.36%)	3 (13.64%)	0.02	1	0.89*
Gastric ulcer	5	4 (80.00%)	1 (20.00%)	1.00	1	1.00**
Gastritis	7	2 (28.57%)	5 (71.43%)	0.0003	1	0.07**

Out of the 36 ureB-positive isolates, 30 (83.3%) were cagA- and vacA-positive (Table 9).

**Table 9:** PCR-based detection of vacA allelic variants in Helicobacter pylori isolates from patients (n = 36) vacA s1: vacuolating cytotoxin A gene, signal region type s1, vacA s2: vacuolating cytotoxin A gene, signal region type s2, vacA m1: vacuolating cytotoxin A gene, mid-region type m1, vacA m2: vacuolating cytotoxin A gene, mid-region type m2.

Endoscopic findings	vacA-positive	vacA s1	vacA s2	vacA m1	vacA m2
Gastric carcinoma	2	Positive	Negative	Positive	Negative
Duodenal ulcer	19	Positive	Negative	Positive	Negative
Gastric ulcer	4	Positive	Negative	Positive	Negative
Gastritis	5	Positive	Negative	Positive	Negative

Out of the 38 culture-positive *H. pylori* isolates, 36 (94.7%) were ureB-positive. The two samples that were negative were discarded from the study.

## Discussion

*H. pylori* is a key pathogen responsible for gastritis, peptic ulcer disease, and gastric malignancies. The prevalence of infection varies according to factors such as age, sex, dietary habits, and genetic predisposition [8].

This study aimed to determine the prevalence of *H. pylori* infection and to investigate associated risk factors, including dietary habits, smoking, and alcohol consumption, among symptomatic patients undergoing UGI endoscopy. Additionally, the pathogenic potential of the isolated *H. pylori* strains was assessed by detecting the presence of cagA and vacA virulence genes.

The prevalence of *H. pylori* infection varies widely between countries and even within regions of the same country, largely due to socioeconomic factors such as poverty, overcrowding, inadequate sanitation, and poor hygiene. Differences are also observed among various ethnic groups within the same population [9].

Namyalo et al. reported that the prevalence of *H. pylori* is around 70% in developing countries, particularly in resource-limited settings such as Africa, whereas in developed countries, it ranges between 25% and 40% [10].

Studies in India reported prevalence rates of 62.7%, 72.8%, 83.5%, and 35.6%, respectively [11-14]. In contrast, our study found an overall prevalence of 15.2%. The relatively low prevalence in our region may reflect a combination of environmental factors, such as socioeconomic status, sanitation, and water supply, as well as host factors including ethnicity, age, and race.

In our study, the highest prevalence (19.3%) was observed in the 41-50-year age group. Vadivel et al. [11], in Chennai, southern India, reported the highest prevalence (58.1%) among 21-39-year-olds, indicating that *H. pylori* prevalence is influenced by topographical, cultural, and behavioral factors. The higher prevalence in the 41-50-year age group in our study could be related to stress and anxiety associated with work, financial obligations, and personal or family responsibilities. Psychological factors such as stress, anxiety, and depression have been shown to significantly influence *H. pylori* infection, as reported by Adlekha et al. [8].

In our study, the prevalence of *H. pylori* infection was higher in males (59.9%) compared to females (40.1%), consistent with the findings of Bharati et al. [9].

Additionally, 25.8% of duodenal ulcer cases in our study were positive for *H. pylori* by culture, a statistically significant association (P < 0.05). This aligns with the study by Cekin et al., in which *H. pylori* was implicated in the pathogenesis of duodenal ulcer disease in 84.9% of patients and identified as the sole causative factor in 44.1% of cases [15].

Among the various risk factors analyzed in this study, only alcohol consumption showed a statistically significant association with *H. pylori* infection (p = 0.0001). Both smoking and alcohol intake are known to compromise gastric mucosal integrity, thereby facilitating colonization by *H. pylori* and contributing to related conditions, including peptic ulcer disease and gastric cancer. This finding is consistent with Bharati et al., who reported a high prevalence of *H. pylori* infection among individuals with lifestyle risk factors, with 62.5% of smokers and 60.7% of alcohol consumers testing positive for the bacterium [9].

*H. pylori* infection typically induces gastric inflammation, and the presence and severity of symptoms often reflect the infection status. A study by Ali et al. demonstrated a significant association between epigastric pain, burning sensations, and *H. pylori* positivity [16]. In the present study, dyspepsia was the most commonly reported symptom among infected patients (24.1%), followed by acid regurgitation (23.2%), indigestion (21.4%), loss of appetite (14.2%), and epigastric tenderness (10.5%).

In this study, the RUT was positive in 177 out of 250 dyspeptic patients (70.8%). Of these RUT-positive patients, 66 (37.3%) were confirmed positive by histological examination. Culture positivity was observed in 38 of the 177 patients (21.5%), of which 36 (94.7%) were also positive by PCR targeting the ureB gene. The relatively low sensitivity of culture may be attributed to multiple factors, including reduced bacterial viability under adverse conditions (e.g., oxygen exposure), low bacterial load in biopsy specimens, the presence of coccoid forms, and the inherent difficulty in cultivating *H. pylori*. Additionally, prior patient use of PPIs or antibiotics can further compromise culture sensitivity [6].

Despite this, culture demonstrated 100% specificity and 100% positive predictive value (PPV) compared to RUT and histology. These findings are consistent with previous studies, which also reported 100% specificity and PPV for culture-based detection of *H. pylori* [17-19]. Similarly, Mutita et al. reported a prevalence of 84.8% using real-time PCR and 19.5% by culture, highlighting the higher sensitivity of molecular methods over conventional culture [20].

Culture positivity rates for *H. pylori* in India exhibit considerable variability. Studies by Sharma et al. and Chyne et al. reported low isolation rates of 4.2% and 3.3%, respectively, whereas Parimala et al. documented a much higher rate of 52.45% [18,21,22]. These differences may be attributed to factors such as the quality of biopsy specimens, bacterial load, the presence of coccoid forms, sample handling, and variations in laboratory techniques, all of which can influence the recovery of viable organisms.

cagA is an important virulence factor that contributes to increased pathogenicity and may be associated with higher risks of gastric carcinoma and treatment resistance among infected patients. *H. pylori* is a genetically diverse pathogen, and the prevalence of cagA-positive strains varies geographically. In the present study, 83.39% of isolates were cagA-positive, a frequency comparable to reports from Mexico, Iran, Iraq, and Bangladesh. This is higher than prevalence rates reported in Palestine, Cuba, Europe, Venezuela, North America, and Pakistan, which are generally around 60%, but lower than rates in Japan and Korea, where cagA positivity exceeds 90% [23-24]. Jeyamani et al. similarly reported that cagPAI prevalence in East Asia was approximately 60%, while in India it ranged as high as 90%, considerably higher than in Western populations [25].

In our study, the prevalence of both cagA and vacA virulence genes was 83.3%, with the highest positivity observed in patients with gastric carcinoma (100%), followed by duodenal ulcer (86.3%), gastric ulcer (80%), and gastritis (71.4%). This high prevalence of cagA is consistent with previous findings, such as those reported by Patra et al., who documented a prevalence rate of 92.3% [26].

Mutita et al. reported that cagA- and vacA-positive *H. pylori* strains accounted for 52.6% and 5.3%, respectively, and were predominantly detected in patients with gastric cancer. cagA-positive strains were found to exhibit stronger adhesion to gastric epithelial cells and induce more pronounced pro-inflammatory responses than cagA-negative strains [20].

In the present study, the vacA s1m1 allele was identified in 83.3% of *H. pylori* samples that were also cagA-positive. *H. pylori* vacA genotypes can be classified into four main combinations: s1m1, s1m2, s2m1 (rare), and s2m2. Among these, s1m1 strains are associated with the highest vacuolating cytotoxin activity in gastric epithelial cells, s1m2 strains with intermediate activity, and s2m2 strains with minimal or no cytotoxicity [3,4]. Specifically, in our positive cases, 80% (72/90) carried the cagA gene. For the signal (s) region of vacA, 98.8% (83/84) of isolates were s1, while 1.2% (1/84) were s2. For the middle (m) region, 63.6% (56/88) were m1, 2.3% (2/88) were m2, and 34.1% (30/88) carried both m1/m2 alleles, consistent with findings reported by Brasil-Costa et al. [27]. Kishk et al. also reported that the vacA+/cagA+ s1m1 genotype is the most frequent and is strongly associated with peptic ulcer disease, gastritis, and gastroesophageal reflux disease [28].

Limitation of the study

While this study focused on the prevalence of *H. pylori* and the distribution of key virulence genes (cagA and vacA), future research could expand the scope to include antimicrobial susceptibility testing.

## Conclusions

The prevalence of *H. pylori* infection observed in this study was lower than that reported in previous Indian studies, with the majority of positive cases associated with duodenal ulcers. Risk factors such as alcohol consumption, smoking, and diabetes were significantly associated with an increased likelihood of infection. Notably, a high proportion of isolates were positive for the cagA gene, which is strongly linked to peptic ulcer disease and carries the potential to contribute to gastric carcinoma. This indicates that the studied population may be at higher risk for severe gastroduodenal complications. Targeted screening of patients presenting with relevant symptoms and associated risk factors could facilitate early identification of cagA-positive strains, enabling timely intervention to prevent disease progression, morbidity, and mortality. Therefore, we recommend routine screening for cagA in patients with *H. pylori* infection to guide appropriate therapy and confirm successful eradication.
